# Feasibility and Safety of Laparoscopic Appendectomy Performed by Residents with No Experience in Open Appendectomy

**DOI:** 10.31662/jmaj.2018-0037

**Published:** 2019-02-01

**Authors:** Ryusei Yamamoto, Yasuji Mokuno, Hideo Matsubara, Hirokazu Kaneko, Shinsuke Iyomasa

**Affiliations:** 1 Department of Surgery, Yachiyo Hospital, Anjo, Japan

**Keywords:** laparoscopic, appendectomy, resident

## Abstract

**Introduction:**

Open appendectomy for acute appendicitis is a common procedure for surgical residents to perform at the beginning of their training. Recently, many programs have moved to laparoscopic appendectomy as the initial training procedure. However, the feasibility and safety of laparoscopic appendectomy for acute appendicitis performed by surgical residents without any experience of open appendectomy remains controversial.

**Methods:**

The records of patients who underwent laparoscopic appendectomy for acute appendicitis between August 2006 and March 2017 were retrospectively reviewed. Patients were assigned to two groups according to whether their procedure was performed by a surgical resident, with no experience of open appendectomy, or a surgical fellow, with adequate open appendectomy experience but no experience with laparoscopic appendectomy.

**Results:**

A total of 130 patients were included. Five residents performed 104 procedures, and three fellows performed 26 procedures. The baseline patient characteristics were comparable between groups. The median operative time was comparable (77.0 min vs. 65.5 min; p = 0.771). There were no significant differences in overall complications; with 14 patients (13%) in the resident group and five patients (19%) in the fellow group experienced complications (p = 0.535). No patient required reoperation, and there were no fatalities. The median length of stay was similar (5.0 days vs. 5.5 days; p = 0.430).

**Conclusions:**

Laparoscopic appendectomy for acute appendicitis is feasible and safe when performed by surgical residents with no prior open appendectomy experience. It may be performed as the first procedure during surgical training with no adverse effect on patients.

## Introduction

Acute appendicitis is one of the most frequent causes of acute abdomen, and it invariably requires an emergent abdominal surgical procedure ^(1), (2)^. Open appendectomy (OA) has long been a standard surgical procedure for acute appendicitis and is a common training procedure at the beginning of a surgical residency ^(3), (4)^.

Laparoscopic procedures, with their minimally invasive nature and favorable outcomes, have been used with increasing frequency in gastroenterological surgery ^(5)^. The number of conventional, open surgeries has markedly reduced, especially for benign disease such as cholecystectomy and appendectomy ^(2), (5)^. Laparoscopic appendectomy (LA) has recently been successfully adopted for patients with acute appendicitis, and OA is now rarely performed without a specific reason ^(2), (6), (7)^. This trend has raised an important issue in surgical education: residents are losing the opportunity for training in open surgeries before they perform the laparoscopic versions. Surgical residents at our institution often perform LA as their first abdominal surgery and their first laparoscopic procedure.

A number of studies have shown LA is feasible and safe when performed by residents, but in most of these studies, surgical residents had prior OA experience before performing their first LA ^(3), (8), (9), (10), (11), (12), (13), (14), (15)^. Although some previous researchers have noted that surgical residents should have experience with OA prior to performing LA, recent articles have indicated residents can perform LA safely, with little OA experience, in either a human or animal model ^(10), (15), (16), (17), (18)^. The feasibility and safety of LA for acute appendicitis performed by surgical residents without OA experience remains unclear.

The aim of this retrospective cohort study is to determine the feasibility and safety of LA as the initial abdominal surgery performed by residents. We accomplish this by comparing the outcomes of LA, performed by residents without OA experience, with those of fellows with antecedent OA experience but without LA experience.

## Materials and Methods

All consecutive records of patients undergoing LA for acute appendicitis between August 2006 and March 2017 at our institution were retrospectively reviewed. Patients undergoing LA for reasons other than acute appendicitis were excluded. Our institution is a medium-sized general hospital, and the department of surgery trains new surgical residents to participate almost every year. Surgical fellows who completed residency at other institutions sometimes transfer in. We first began performing LA for acute appendicitis in August 2006; it is considered the first-line treatment for acute appendicitis unless there are exceptional circumstances. Most surgeons performing LA are surgical residents or fellows. The ethics board of Yachiyo Hospital, Japan, approved this study; however, our institutional review board does not issue an individual approval code for a retrospective cohort study.

### Participants

Patients undergoing LA for acute appendicitis were assigned to two groups: those whose procedures were performed by surgical residents (postgraduate years 1–3), and those whose procedures were performed by surgical fellows (3 years of residency completed). Five surgical residents were eligible. These residents performed LA as their first abdominal procedure and first endoscopic procedure; none had OA experience. Three surgical fellows were eligible. They had adequate OA experience and some experience with other endoscopic and open abdominal surgeries, but they had never performed LA before coming to our facility. Patients who underwent LA performed by a surgical resident with concurrent experience in LA and OA, a surgical fellow with antecedent experience in LA, or the attending surgeon were excluded from analysis.

### Surgical procedure

Three trocars were placed with a rigid laparoscope for visualization. The mesoappendix was divided using laparoscopic coagulating shears, and the appendix was resected using a loop ligature or endostapler. The surgeon decided whether to insert drainage tubes in patients with complicated appendicitis. All surgical residents worked under the guidance and supervision of board-certified attending surgeons; however, residents performed the whole procedure as the operator. The attending surgeons did not perform any part of the procedure unless the residents encountered severe technical difficulties.

### Outcomes

The outcomes of interest were complications, operative time, blood loss, conversion to OA, reoperation within 30 postoperative days, length of hospital stay, and mortality. The results were compared according to the operator type: resident or fellow. The analysis of complications incorporated any morbidity that occurred after LA.

### Statistical analysis

All statistical analyses were conducted using SPSS statistical software (version 20.0; IBM Corporation, Armonk, NY). Differences in baseline characteristics and outcomes between groups were analyzed using the Mann–Whitney U test for continuous variables and Fisher’s exact test, or the chi-square test, for categorical variables. A two-sided p value of <0.05 was considered statistically significant.

## Results

**Figure 1.**
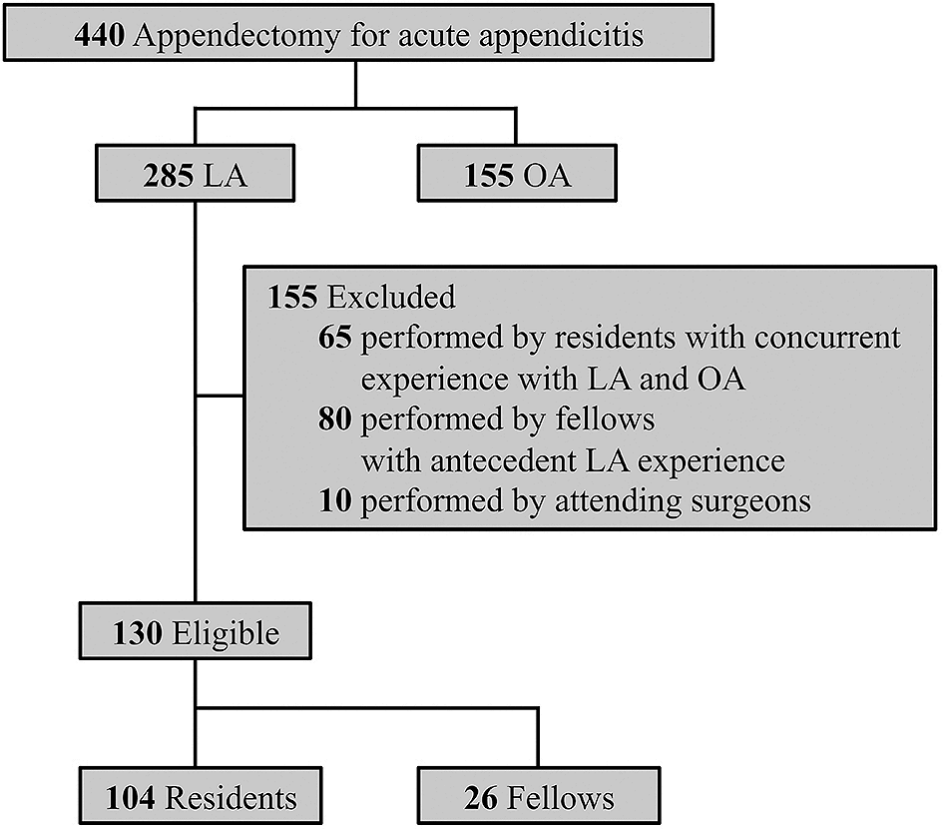
Flow diagram. LA, laparoscopic appendectomy; OA, open appendectomy.

Of the 285 patients who underwent LA for acute appendicitis during the study period, 130 satisfied the inclusion criteria. A resident performed LA on 104 patients, and a fellow performed LA on 26. Of the 155 patients excluded, 65 underwent LA performed by a resident with concurrent experience with LA and OA, 80 had their procedures performed by surgical fellows with antecedent LA experience, and 10 had their procedures performed by the attending surgeon (Figure. 1).

The baseline patient characteristics are shown in Table 1. Both groups were comparable in terms of age, sex, body mass index, American Society of Anesthesiologists classification, and preoperative white blood cell count. The fellow group had slightly higher levels of C-reactive protein (CRP). The appendix pathology did not differ between groups; the incidence of gangrenous appendicitis, including perforated appendicitis, was 55% (57 patients) in the resident group and 54% (14 patients) in the fellow group. The incidence of complicated appendicitis, including intra-abdominal abscess with or without perforation, was 24% and 38%; this difference was not statistically significant.

**Table 1.** Baseline Patient Characteristics.

	Resident Surgeon	Fellow Surgeon	*p* Value
	n (%)	n (%)	
Patients	104	26	
Age, years^†^	37.0 (19.0–47.5)	40.5 (21.0–63.0)	0.381
Sex, male	72 (69%)	15 (58%)	0.351
BMI (kg/m^2^)^ †^	22.0 (20.0–25.1)	21.8 (19.9–23.8)	0.610
ASA			0.295
1	82 (79%)	17 (65%)	
2	15 (14%)	7 (27%)	
3	7 (7%)	2 (8%)	
WBC (10^3^/µL) ^†^	14.0 (11.2–16.9)	13.2 (9.3–17.3)	0.481
CRP (mg/dL) ^†^	1.42 (0.24–5.00)	2.85 (0.84–13.45)	0.038
Complicated appendicitis	25 (24%)	10 (38%)	0.146
Pathology			0.420
Catarrhal	6 (6%)	0 (0%)	
Phlegmonous	41 (39%)	12 (46%)	
Gangrenous	57 (55%)	14 (54%)	

^†^data listed as median (interquartile range). BMI, body mass index; ASA, American Society of Anesthesiologists classification; WBC, white blood cell count; CRP, C-reactive protein.

**Figure 2.**
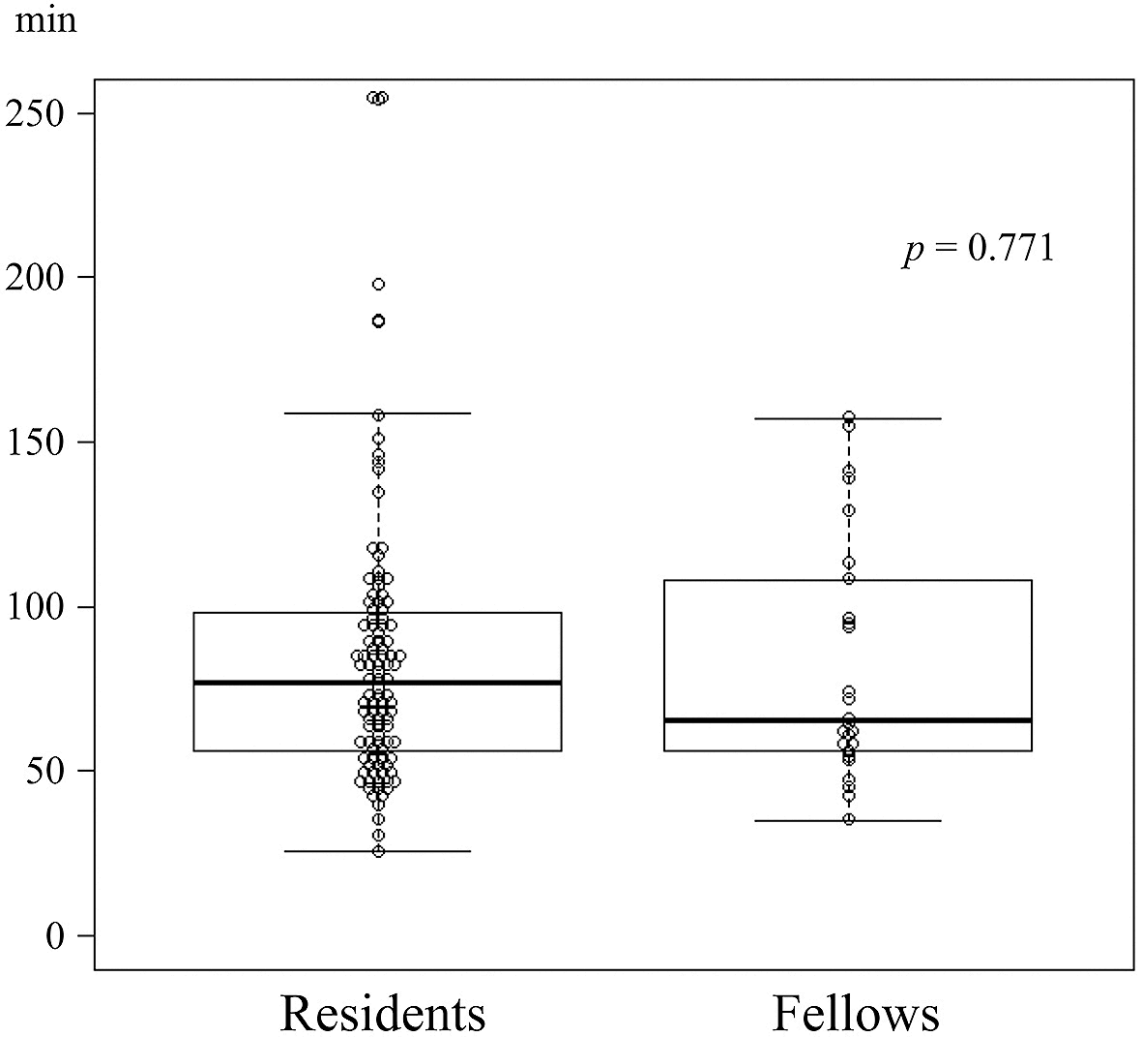
Operative time for LA is comparable between the groups (median: 77.0 min vs. 65.5 min; p = 0.771). LA, laparoscopic appendectomy.

The LA outcomes according to group are shown in Table 2. The median operative time was comparable (77.0 min vs. 65.5 min; p = 0.771) (Figure 2), and there was no difference in the amount of blood loss (5 g vs. 5 g; p = 0.572). A single patient in each group required conversion to OA (1% vs. 4%; p = 0.361). The indication for conversion in the patient in the resident group was bleeding from the mesoappendix, and the indication for the patient in the fellow group was severe inflammatory adhesions. There were no significant differences in overall complications; fourteen patients (13%) in the resident group and five patients (19%) in the fellow group experienced complications. In the resident group, intra-abdominal abscess occurred in five patients (5%), ileus in seven patients (7%), diarrhea in one patient, and rhabdomyolysis in one patient; no patients experienced wound infection. In the fellow group, intra-abdominal abscess occurred in three patients (12%), wound infection in one patient, and diarrhea in one patient. No patient required reoperation, and there was no mortality. The median length of hospital stay was comparable (5.0 days vs. 5.5 days; p = 0.430).

**Table 2.** Outcomes of Laparoscopic Appendectomy.

	Resident Surgeon	Fellow Surgeon	*p* Value
	n (%)	n (%)	
Length of stay, days^†^	5.0 (4.0–7.0)	5.5 (4.3–9.5)	0.430
Operative time, min^†^	77.0 (57–98)	65.5 (57–105)	0.771
Blood loss, g	5 (3–10)	5 (1–14)	0.572
Overall complications	14 (13%)	5 (19%)	0.535
Wound infection	0	1 (4%)	0.200
Intra-abdominal abscess	5 (5%)	3 (12%)	0.198
Ileus	7 (7%)	0	0.344
Clavien–Dindo classification			0.108
1	8 (8%)	2 (8%)	
2	4 (4%)	0	
3a	2 (2%)	3 (12%)	
Conversion	1 (1%)	1 (4%)	0.361
Reoperation	0	0	1.000
In-hospital mortality	0	0	1.000

^†^ data listed as median (interquartile range).

## Discussion

Our study shows that LA performed by residents with no prior OA experience yields good surgical outcomes. We found no significant difference between these residents and fellows with adequate antecedent OA experience. Therefore, we conclude that LA is a suitable initial operation for surgical residents under appropriate supervision.

Our finding that resident-performed LA does not have a higher incidence of overall complications than fellow-performed LA is consistent with other studies. The overall complication rate after LA performed by residents is 13 percent, comparable to the rate when LA is performed by fellows and similar to the previously reported complication rate of 4%–17% for residents performing LA for acute appendicitis ^(3), (14), (15)^. In our study, resident-performed LA resulted in no serious morbidity, reoperation, or mortality. Five patients did develop an intra-abdominal abscess, two of whom required percutaneous drainage, but the other three patients were cured with antibiotics alone. Postoperative ileus developed in seven patients: a single patient required long term nil per os status without a nasogastric tube and was hospitalized for one month; the others were cured with a few days of fasting. No patients experienced wound infection. These findings indicate LA’s safety for acute appendicitis performed by surgical residents with no prior experience of OA. One case required conversion due to bleeding from the mesoappendix. Surgical residents without open surgery experience may not accomplish the laparotomy if conversion is required; therefore, an attending surgeon should be involved in the surgery.

We did not find that LA performed by our resident group results in a longer length of stay than LA performed by our fellow group. Our finding of 5 days in the resident group and 5.5 days in the fellow group is slightly longer than reported in previous studies ^(3), (14), (15)^. This may be a consequence of the Japanese medical insurance system, and the clinical pathway of our institution, which required hospitalization for six days as recently as 2016.

Our results could be explained by laparoscopic surgery’s characteristics. Due to the benign nature of acute appendicitis, OA is usually undertaken through a small laparotomy, which sometimes makes it difficult for surgeons to share the view of the surgical field. In contrast, LA allows for sharing of a clear, multiangle surgical field on the monitor. The attending surgeons can observe the procedure and provide appropriate, timely advice. All residents at our institution review operating videos with the attending surgeons immediately after any procedure, allowing them to observe many more cases than they actually perform.

Some previous studies that include surgical residents, with either antecedent experience of OA or laparoscopic training, suggest that the learning curve of LA is 20–30 cases. In our study, the mean number of LAs performed by each resident is 21. The operative time of 77 min is similar to the operative time reported in previous studies describing 67 to 88 min during the learning curve ^(10), (11)^. This may be because our residents review operation videos, as described above, increasing their exposure to different types of surgery. In five cases, the extremely severe extent of inflammation and adhesion resulted in very long operation times. Although residents completed each LA, two patients experienced a postoperative intra-abdominal abscess; there was no other postoperative complication.

Because of the retrospective nature of this cohort study, selection bias may exist. In fact, the patients in our fellow group have higher CRP levels. However, the other baseline patient characteristics are comparable. The extent of inflammation present with appendicitis is indicated by the difficulty of the surgical procedure and the postoperative outcomes. In our study, the proportion of gangrenous appendicitis, including perforated appendicitis, is comparable between groups, and the proportion of complicated appendicitis, while slightly higher in the fellow group, is not significantly different. The 55% rate of gangrenous appendicitis in our study, reported as 14%–28%, is greater than the rates described in previous studies ^(3), (14), (15)^. In other words, the degree of difficulty involved in the appendectomies in our study may be higher than the degree in previous studies.

Other potential study limitations include the fact that our sample size for fellow-performed LA is small. Each fellow performed a mean of 8.7 LAs. The outcomes in the fellow group may be improved if fellows performed more LAs. Despite these limitations, we are convinced that our analysis demonstrates LA is a safe initial abdominal laparoscopic operation for trainees.

We conclude that LA for acute appendicitis is safe and feasible when performed by surgical residents without prior OA experience. Performing LA as the initial abdominal surgery during training, under the guidance of an experienced staff surgeon, does not adversely affect the patient.

## Article Information

### Conflicts of Interest

None

### Author Contributions

All authors contributed to the concept of this article. RY drafted the manuscript; YM provided revisions; and HM, HK, and SI supervised the writing of the article. All authors participated in interpreting the results and writing the article. All authors approved the final version of the manuscript.

### Approval by Institutional Review Board (IRB)

The ethics board of Yachiyo Hospital, Japan approved this study. Our institutional review board does not issue individual approval codes to retrospective cohort studies.
